# Interplay wellbeing framework: a collaborative methodology ‘bringing together stories and numbers’ to quantify Aboriginal cultural values in remote Australia

**DOI:** 10.1186/s12939-017-0563-5

**Published:** 2017-05-03

**Authors:** Sheree Cairney, Tammy Abbott, Stephen Quinn, Jessica Yamaguchi, Byron Wilson, John Wakerman

**Affiliations:** 1 Centre for Remote Health, a Joint Centre of Flinders University and Charles Darwin University, Alice Springs, NT Australia; 2 Ninti One Limited and the Cooperative Research Centre for Remote Economic Participation (CRC-REP), Alice Springs, NT Australia; 3 Swinburne University, Melbourne, VIC Australia; 4 Menzies School of Health Research, Charles Darwin University, Darwin, NT Australia; 5 Policy, Analysis and Evaluation Division, Australian Government Department of the Prime Minister and Cabinet, Canberra, ACT Australia

**Keywords:** Wellbeing, Aboriginal, Indigenous, Culture, Empowerment, Framework, Policy, Aboriginal literacy, Stories

## Abstract

**Background:**

Wellbeing has been difficult to understand, measure and strengthen for Aboriginal people in remote Australia. Part of the challenge has been genuinely involving community members and incorporating their values and priorities into assessment and policy. Taking a ‘shared space’ collaborative approach between remote Aboriginal communities, governments and scientists, we merged Aboriginal knowledge with western science – by bringing together stories and numbers. This research aims to statistically validate the holistic Interplay Wellbeing Framework and Survey that bring together Aboriginal-identified priorities of culture, empowerment and community with government priorities including education, employment and health.

**Method:**

Quantitative survey data were collected from a cohort of 842 Aboriginal people aged 15-34 years, recruited from four different Aboriginal communities in remote Australia. Aboriginal community researchers designed and administered the survey.

**Results:**

Structural equation modeling showed good fit statistics (*χ/df* = 2.69, CFI = 0.95 and RMSEA = 0.045) confirming the holistic nature of the Interplay Wellbeing Framework. The strongest direct impacts on wellbeing were ‘social and emotional wellbeing’ (*r* = 0.23; *p* < 0.001), ‘English literacy and numeracy’ (*r* = 0.15; *p* < 0.001), ‘Aboriginal literacy’ (*r* = 0.14; *p* < 0.001), ‘substances’ (lack thereof; *r* = 0.13; *p* = 0.003), ‘work’ (*r* = 0.12; *p* = 0.02) and ‘community’ (*r* = 0.08; *p* = 0.05). Correlation analyses suggested cultural factors have indirect impacts on wellbeing, such as through Aboriginal literacy. All cultural variables correlated highly with each other, and with empowerment and community. Empowerment also correlated highly with all education and work variables. ‘Substances’ (lack thereof) was linked with positive outcomes across culture, education and work. Specific interrelationships will be explored in detail separately.

**Conclusion:**

The Interplay Wellbeing Framework and Survey were statistically validated as a collaborative approach to assessing wellbeing that is inclusive of other cultural worldviews, values and practices. New community-derived social and cultural indicators were established, contributing valuable insight to psychometric assessment across cultures. These analyses confirm that culture, empowerment and community play key roles in the interplay with education, employment and health, as part of a holistic and quantifiable system of wellbeing. This research supports the holistic concept of wellbeing confirming that everything is interrelated and needs to be considered at the ‘whole of system’ level in policy approaches.

## Background

### Wellbeing as a measure of success

The way that progress or success is defined and measured by any given society both represents *and* drives its values and goals [1]. Modern societies across the world have prioritized economic markers of success, but more recently a broader concept of wellbeing has emerged that encompasses the many facets that influence one’s ‘quality of life’ [1]. Depending on societal and cultural priorities, this can include combinations of education or learning, livelihoods, health, environmental, social and cultural factors. The term ‘happiness’ has also become a popularized substitution for ‘wellbeing’ [1].

The way in which governance bodies define and measure wellbeing is therefore not only an expression of a society’s values and goals, but also has a strong influence on peoples’ daily lives through driving government policy. The way we define and measure wellbeing therefore matters a great deal – it shapes our national and societal values, knowledge and pathways.

The challenge for governments is to design national and localised approaches to wellbeing that best accommodate the needs of diverse or displaced populations [2]. Inevitably national approaches prioritise the ‘mainstream’, or those who share the values and goals of their external governance structures.

### Aboriginal and Torres strait islander wellbeing in remote Australia

Those most negatively affected by this approach - often referred to as ‘minority’ groups – are those living within governance structures whose values and goals are most divergent from their own [2]. That is, one ‘worldview’ and its associated law and order are imposed on another by a more powerful governing body. One of the strongest examples of this globally is Aboriginal and Torres Strait Islander people living today in Australia’s most remote regions [2]. Many continue to live on their inherited ancestral lands, with daily cultural practices based on ancient cultural law and protocol that have been passed down through generations for tens of thousands of years, and are vastly different from modern mainstream practices [3–6]. This is the most extreme end of a continuum for Australia’s Aboriginal and Torres Strait Islander population whose experiences range between the polarities of living on their ancestrally inherited lands (‘on country’) in remarkably preserved ancient cultures, to those more integrated to the ‘mainstream’ through several generations of colonial influence - often under traumatically enforced assimilation policies.

Aboriginal and Torres Strait Islander people in Australia continue to be ranked amongst the poorest in the nation on measures used to assess and compare wellbeing that include education outcomes, financial income and health outcomes [2, 7–11]. Incarceration rates and life expectancy differ startlingly to national standards [7]. Reducing these inequities has been the focus of national ‘closing the gap’ policies and substantial investment over many decades [9, 12] with little improvement recorded, prompting the need for a fresh approach [10, 11, 13].

A cultural bias or cultural dependence is created when national policy and measures of success represent a majority, and in doing so, exclude the needs and values of diverse cultural groups [2]. In Australia, this has been challenged as a ‘deficit’ approach based on its underlying assumption that Aboriginal people will experience a better quality of life if they adopt mainstream values and practices, and are considered to ‘fail’ when they do not [13]. At a fundamental level is a failure of governments to genuinely involve Aboriginal and Torres Strait Islander people in their own solutions, and acknowledge and accommodate Aboriginal cultural values and priorities – thus creating an externally imposed approach to wellbeing [13].

Wellbeing frameworks represent a holistic approach that incorporates and expands upon the global recognition that ‘social determinants’ account for a substantial portion of health outcomes, particularly in poorer communities [2, 8, 14]. Ironically, holistic wellbeing approaches to societal progress or success, such as those now pursued globally [1], align more closely with those of Aboriginal cultures as acknowledged by the Australian Government in their National Aboriginal Health Strategy in 1989 [12] and in the ‘Social and Emotional Wellbeing’ approach to Aboriginal mental health, defined by the Healing Foundation as:

“…*our feeling of being healthy on a physical, spiritual, emotional and social level. It is a state where individuals and communities are strong, proud, happy and healthy. It includes being able to adapt to daily challenges while leading a fulfilling life. For Aboriginal and Torres Strait Islander people land, family and spirituality can also be considered central to wellbeing.”* [15].

However, integrating these holistic concepts into existing systems of monitoring, evaluation policy and service delivery represents a substantial paradigm shift and methodological challenge, with limited progress [2, 7–11, 13, 14].

Two recent comprehensive reviews revealed significant shortcomings in the availability of appropriate psychometric assessments of wellbeing or quality of life for Indigenous people and identified a clear need to develop new indicators and assessments designed specifically with and for this population [16, 17].

### ‘Stories and numbers’

Aboriginal cultural values and practices are grounded in spiritual connection to the land, or ‘country’ and practiced as language, law, kinship/family systems and ceremony [3]. Beliefs are holistic with everything being interconnected [4, 5]. People exist as part of an interrelated continuum with all of nature – including plants, animals and the land. People are borne into known and maintained relationships with all living things defined by kinship systems, totems and stories. Aboriginal culture is placed in sophisticated ancient systems of knowledge, law, science and research [18–20]. However, because Aboriginal knowledge is transmitted orally – through stories – much of this knowledge is not physically recorded, or is lost through the impact of colonization [2].

The reliance of western systems of knowledge and governance on empirical evidence and ‘numbers’ has posed substantial challenges worldwide in areas such as culture and arts [21]. While recognized as essential aspects of wellbeing, their value is difficult to ascertain – and hence for governments to justify resource allocation – as they do not readily lend themselves to quantification, particularly for comparative analyses [22, 23]. More innovative approaches to assessment that combine qualitative and quantitative information are needed to represent the value – in all its richness and complexity - of culture and arts in current political systems.

The major challenge is for the ownership and detail (i.e. indicators) of wellbeing frameworks to maintain integrity of values and worldviews across cultural, political and scientific interfaces. Partnership and collaboration between these sectors are necessary to achieve genuine change. Bridging worldviews with such vast differences in conceptual thinking and ways of working represents a substantial, but not impossible challenge. Transformative progress demands replacing the status quo for novel, inclusive and more empowering approaches.

This research aims to improve wellbeing for Aboriginal groups in remote Australia by quantifying their values and priorities in a framework to inform government policy. This represents a novel and inclusive approach and methodology that have likely relevance for Torres Strait Islander groups and other Australian Aboriginal groups in urban communities, as well as other minority and culturally diverse groups globally, although this is beyond the scope of the current paper.

### The Interplay project

The Interplay research project aimed to develop and validate – both culturally and scientifically - a framework to quantify a holistic concept of wellbeing for Aboriginal and Torres Strait Islander people in remote Australia, for the purpose of informing policy and practice [24]. The development process is reported in detail elsewhere with the three main stages summarised below given their direct relevance to the current analysis [2, 6, 24, 25].

**Fig. 1:**
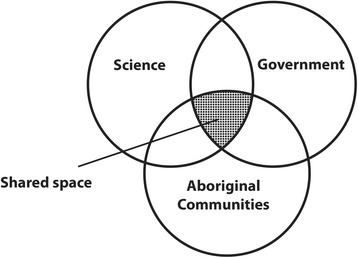
The ‘Shared Space’ approach to working collaboratively between communities, government and scientists
  As part of the shared space approach, 42 local Aboriginal community researchers were recruited, trained and employed on the project to contribute to the design, data collection and interpretation. The key to the success of this approach lay in the depth of knowledge sharing between the groups – afforded through substantial time and discussion effort. For example, when Aboriginal researchers and scientists differed on their preference for the wording of a question or the nature of response options, discussions were then held with each group describing why they held such preferences. When each group understood the needs of the other, they were able to appreciate them and identify which parts of their own standpoint were flexible and which must be maintained, and shared agreements were made in each case towards common goals. Aboriginal community researchers stated they had never before been involved in the development stages of the research, and never before had they understood it so much. They felt they were part of the ‘thinking as well as the doing’, with one person stating, “I’m having so much fun doing this!” [6].

1. *Literature Review*
  First, a literature review was conducted to inform the framework [2], generating the following recommendations: (1) the genuine involvement of Aboriginal and Torres Strait Islander people in all stages of the research and their perspectives represented; (2) taking a strengths-based approach; (3) focusing on interrelationships; (4) including: culture, kinship, land and spirituality; control and empowerment; healthy, safe and inclusive communities and resilience and (5) taking broader and more flexible definitions of education, employment and health. These recommendations were then upheld and applied through all subsequent stages of the research. In 2013, this was in the top 10 of most downloaded documents for Australian Policy Online, confirming its relevance for national policy.

2. *The ‘Shared Space’ Approach to Working Collaboratively*
  Second, a ‘shared space’ approach to working collaboratively was developed and applied whereby each component of the research including developing aims, design, implementation, interpretation, knowledge translation and communication tools - was conducted in the conceptual ‘shared space’ between the project’s three core partner areas of community, government and science (Fig. 1) [6]. All decisions, actions and communications were conducted within this space. Importantly, the criterion was for every part of the research to make sense and be accessible to each of these three groups. This approach is unique in that it is both ground-up (or ‘grass roots’) and top-down and ensures strong foundations in science, community development and policy impact, with end-users serving as contributors to the project [6]. It also represents a program of capacity development.

3. *Extensive ‘Grass-roots’ Community Consultation*
  Third, an extensive national ‘grass-roots’ consultation process was conducted with remote Aboriginal communities nationally over 3 years from 2011 to 2013, with 242 people engaged through 17 workshops and a series of meetings, interviews and community visits [25]. Importantly, a thematic analysis of these qualitative data identified that – despite significant cultural diversity – three core priorities were consistent for Aboriginal people from remote communities nationally. These were: culture, empowerment and community [25].

### The Interplay Wellbeing Framework

**Fig. 2:**
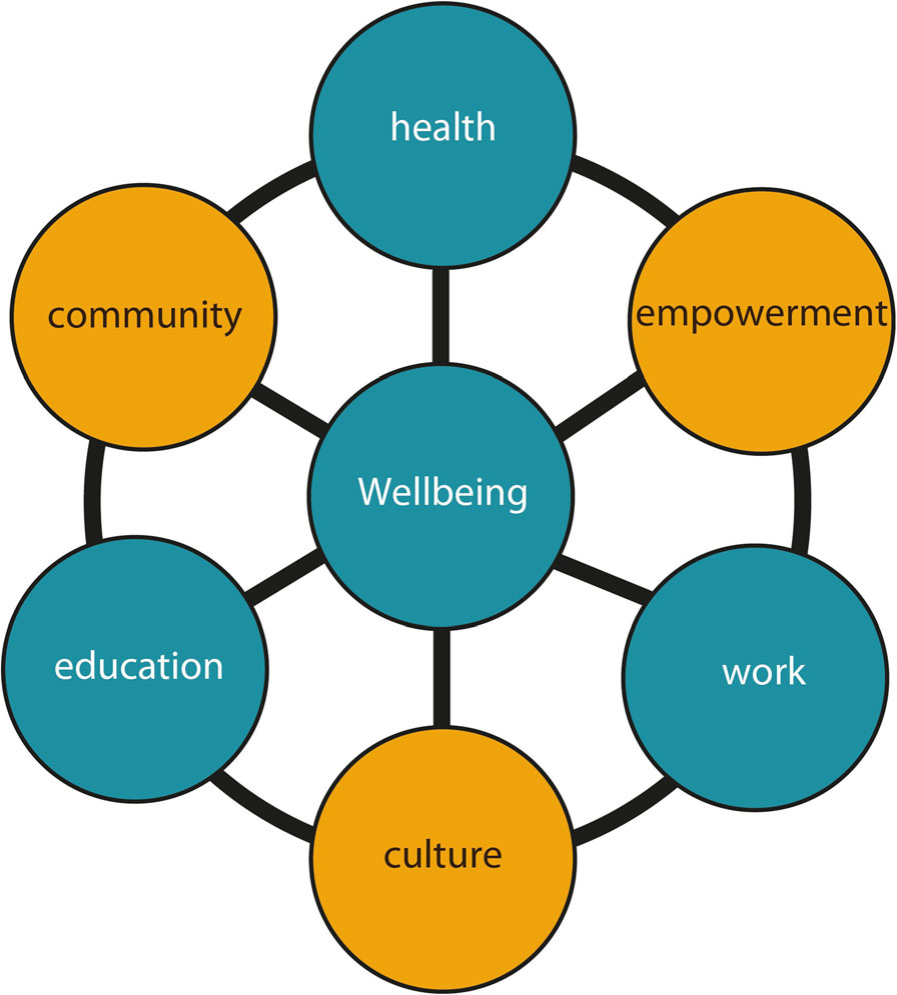
The Interplay Wellbeing Framework

The ‘Interplay Wellbeing Framework’ was then developed bringing together government priorities (based on national ‘Closing the Gap’ policies) of education, employment and health, together with community identified priorities of culture, empowerment and community (Fig. 2).

**Fig. 3:**
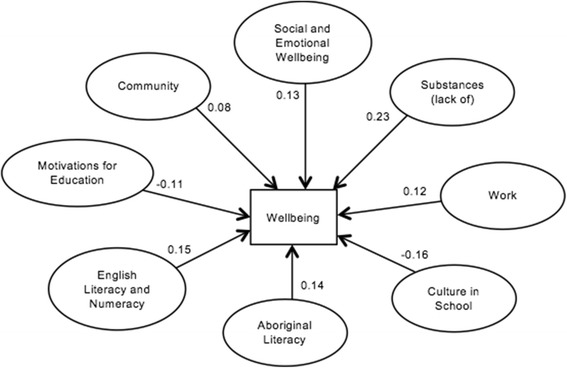
Direct relationships between latent concepts and wellbeing that are statistically signifiant (*P* < 0.05)

The consultation process was also used to inform the definition and measurement of the constructs of culture, empowerment and community, and provide more contextually relevant definitions of the government priority areas of education, employment and health [25]. For example, the definition of education was broadened to include the teaching and learning that happens outside of the school, as part of cultural learning and development. Employment was also broadened to accommodate the non-paid work that is part of daily cultural and family obligations, and often prioritised over paid work due to its more personally beneficial impacts on wellbeing [25].

These definitions and processes were also applied to develop a survey - based on the Interplay Wellbeing Framework and described in the methods section below - to generate quantifiable measures of the values and priorities identified by Aboriginal communities in remote Australia. In a mixed methods design, participatory action research was therefore applied, using qualitative data (e.g., ‘stories’) [26] to inform the design of the Interplay Wellbeing Framework and accompanying survey.

The current paper outlines a statistical validation of the holistic Interplay Wellbeing Framework and accompanying survey to quantify wellbeing and the interrelationships or ‘interplay’ between its underpinning indicators. Essentially it tests the hypothesis that everything within the framework is related to everything else. Specific statistical interrelationships and qualitative data will be explored in subsequent reports, and longitudinal data are currently being collected against the framework, also to be reported subsequently.

## Methods

### Approach

This research is part of the ‘Interplay Project’ for the Cooperative Research Centre for Remote Economic Participation (CRC-REP) managed by Ninti One Ltd. A multidisciplinary participatory action approach [27] embedded end-users in the project from its onset as research partners (including remote community organisations and the Australian Government’s Department of Prime Minister and Cabinet) and through a national advisory group [24]. Aboriginal and Torres Strait Islander people have ownership over the Interplay project research at various levels. Both the management and Advisory Groups comprise approximately 50% Aboriginal and Torres Strait Islander representation, 44 of 47 team members (mostly field researchers) (93%) and two authors are Aboriginal. Field researchers were employed through the Ninti One Aboriginal Community Researcher program [6]. Our approach to community partnerships was informed by Michael LaFlamme’s *Learning Journeys: seven steps to stronger remote communities*, where the right people are identified at the community level to engage in a two-way learning process with outsiders towards achieving change through building empowerment and capacity in remote communities [28].

### Study design

A mixed methods research design was applied, with qualitative methods informing the development of the Interplay Wellbeing Framework and survey that were, in turn, used to collect quantitative data. Additional qualitative data were also collected against the Interplay Wellbeing Framework, to be reported in a separate analysis.

### Community selection

This research was designed to produce reliable estimates and indicators of wellbeing for Aboriginal and Torres Strait Islander people living in either ‘remote’ or ‘very remote’ areas, based on the Australian Standard Geographical Classification (ASGC) categories used by the Australian Bureau of Statistics (ABS) [7]. We approached sampling as if taking a representative sample, and then scaled back based on the limitations of project resources. With 838 discrete Aboriginal and Torres Strait Islander communities in remote and very remote Australia, participation of 30 communities would justify a representative sample. Project advisors preferred a case-study approach prioritising ‘depth over breadth’ whereby deeper consultation with fewer communities would enable a more profound understanding of strengths, issues and challenges. This approach and knowledge gained could then be expanded to understand how it relates more broadly. This was preferred over a ‘one size fits all’ approach to national Aboriginal and Torres Strait Islander issues that has been criticised for being ‘top down’ and not accommodating grass-roots needs and beliefs, or cultural diversity [2].

Community self-selection occurred whereby the Interplay project was promoted through our networks, and community groups then approached the research team expressing their interest to be involved. This process established a strong sense of ownership and empowerment for the community groups, who therefore initiated the research in their communities and were then heavily involved in its development. Engagement began with eight remote communities across Northern Territory, Western Australia and South Australia who contributed to research development [6] and later consolidated with four communities who continued to participate in the research. Community selection was therefore based on a combination of sampling methods, established relationships with partner organisations within communities, and community self-selection, whereby the community could use the research to address a locally identified priority that could be addressed through wellbeing assessment [24].

Four Aboriginal communities across the Northern Territory and Western Australia participated in the research, with two classified as ‘remote’ and two as ‘very remote’ (summarised in this article as ‘remote’) [7]. Participating communities represented diversity across geography, culture, language, population size and characteristics of infrastructure and service delivery.

### Participants

Aboriginal and Torres Strait Islander people comprise the Indigenous or First Australians, however all of these terms are collective and represent many different language and cultural groups. As no Torres Strait Islanders participated in this research, we refer in this report to participants collectively as ‘Aboriginal’. However, they represent many different clan and language groups.

Surveys were collected from 917 Aboriginal people aged 15-34 years from 2014 to 2015. This age range was selected as that closest to when individuals undergo the life course transition from education through to employment, to ensure meaningful outcomes were delivered within the project’s 6-year lifecycle. A total of 73 participants were excluded based on their age >34 years, and a further 2 who were non-Aboriginal. The final cohort comprised of 842 participants (mean age = 25.2 years, SD 5.3; 352 Males, 489 Females). While sample sizes are generally not used in structural equation modeling (applied here), Bentler and Chou [29] suggest a ratio of five cases per variable where latent variables have multiple indicators. Our final model consists of approximately 40 items (see below) and our sample size of 842 therefore provides 21 observations per item, providing sufficient power for this analysis.

### Procedure

#### Interplay survey

The Interplay survey was designed to collect locally relevant, contextual, quantitative data based on the Interplay Wellbeing Framework. A rigorous process was undertaken to develop a survey with cultural and scientific validity [24]. Firstly, a comprehensive review was undertaken of all surveys related to wellbeing or any of its subcomponents that have previously been developed and validated (both scientifically and culturally) for use with Aboriginal and Torres Strait Islander people. Secondly, all questions and strategies used in comparable research were reviewed, including the Longitudinal Study of Indigenous Children [30, 31], the West Australian Aboriginal Child Health Survey [32], the National Aboriginal and Torres Strait Islander Social Survey [33], Strong Souls [34], the Global Empowerment Measure (GEM) [35], the SeIQOL [36], the National Indigenous Languages Survey (NILS) [37], the Caring for Country questionnaire [38, 39] and the SF36 [40]. The Ngurru-Kurlu was also used to inform cultural indicators [3]. Outcomes from these processes were reviewed in a workshop with Aboriginal community researchers from the four participating remote communities, who worked with the research team to draft a tailored survey based on the Interplay Wellbeing Framework. This involved modifying existing survey items as necessary, and developing new items to represent areas of the framework where quantifiable measures have not been established. For example, the importance of culture is clear, but robust means for its measurement did not exist.

The final survey questions reflect in-depth discussions between Aboriginal community researchers, with their knowledge of everyday scenarios faced by people in remote communities, and scientists, with their knowledge about how to represent these scenarios in statistically sound survey questions. Considerable time was afforded to these discussions, with much emphasis on the wording of every single question to ensure that the meaning held true; both in the cross-cultural setting, when translated into local languages, and in different communities nationally. This process was essentially about translating the ‘stories’ into ‘numbers’. Aboriginal community researchers opted to run the survey in English for consistency but translate locally as required. After several waves of reviewing and refining the survey with Aboriginal community researchers and other team members, it was pilot-tested with positive outcomes in two remote communities and considered field ready. The final survey was administered on computer tablets using isurvey software by local Aboriginal community researchers and took approximately 45-60 min to complete.

#### Measures

The Interplay survey was administered to participants to assess self-reported indicators of wellbeing [24]. The six domains from the Interplay Wellbeing Framework shown in Fig. 2 (culture, empowerment, community, education, work and health) were comprised of sub-domains represented by survey items that are outlined in Table 1.

**Table 1:** Domains and sub-domains represented in the Interplay survey

Domains	Sub-domains
Culture	Language, country, law, ceremony, family, importance of culture, practicing culture, culture in school
Community	Leadership, safety, connectedness, trust and respect, services
Empowerment	Inclusiveness, mobility, resilience, self-efficacy, identity, agency, hope
Education	Achievements/outcomes, English literacy and numeracy, focus, motivations, barriers, pathways to work
Work	Paid job, volunteer work, cultural and family work, pathways from education, culture at work, motivations, barriers, work life balance, value/meaning in work
Health	Nutrition, food security, exercise, substance use, anxiety, depression, medical conditions, physical health, dental health, health services, barriers
Wellbeing	Now, past, future

Most survey questions were modelled with a 5-point Likert scale ranging from 0 (Not at all), 2 (Sometimes) 4 (Lots) [24]. Higher scores indicate higher levels of the domain, or latent trait. Some items such as *Motivations for education* were dichotomous indicating agreement or not.

#### Statistical analysis

Descriptive statistics, means, standard deviation and standardized Cronbach alphas were reported using Stata 14.1 (StataCorp, College Station, Texas). Missing data were calculated using multiple imputations, redrawing 11 samples, and taking the median as the most likely value. To reinforce a strength-based approach and dialogue, all items were recoded so that higher response values represent more positive impacts on wellbeing. The primary outcome of wellbeing was self-assessed and measured on a ten point visual analogue scale, based on Cantril's Self-Anchoring Scale. Exploratory factor analysis was conducted using Statistical Packages for Social Science (SPSS; IBM Corporation, Meadville, Pennsylvania) on the six a priori identified domains within the Interplay Wellbeing Framework (Fig. 2), using maximum likelihood estimation, with Promax rotation. Factors were selected with eigenvalues greater than one. Structural equation modeling using AMOS v22 (IBM Corporation, Meadville, Pennsylvania) was then used to assess the relationships between the domains and wellbeing. Items within the same domains concept were allowed to correlate to improve model fit. Table 2 shows domains and sub-domains (latent traits or survey items) retained in the final analysis. The model was assessed using the comparative fit index (CFI), the root mean square error of approximation (RMSEA) to account for model complexity and the maximum likelihood chi-square/degrees of freedom. The four communities were defined as ‘remote’ or ‘very remote’ based on the ASGC categories used by the Australian Bureau of Statistics (ABS) [7] and measurement invariance was investigated according to community remoteness. All confidence intervals reported are 95% and a *p*-value (two-tailed) ≤ 0.05 was deemed to be statistically significant.

**Table 2:** Domains and sub-domains retained in final analyses

Latent concept	Sub-statement (survey item)
Importance of culture	Law
	Ceremony
Practice culture	Caring for country
	Hunting / food sources
Culture in school	Learned about my culture
	Learn in first language
	Community support of school
Aboriginal literacy	Read Aboriginal language
	Write Aboriginal language
Empowerment	Resilience
	Self-efficacy
	Identity
Community	Feels safe
	Works well together
	Trust and respect
Motivations for Education	Improve English skills
	Learn new things
	Improve confidence
English literacy and Numeracy	Speak English
	Read English
	Write English
	Understand numbers
	Add and subtract
Work	Paid work
	Voluntary work
	Study/education
General health	Normal activities
	Work or study
	Energy levels
	Socialising
Social and emotional wellbeing	Worries-hard to breath
	Worries-dizzy
	Worries-shaky
	Too many bad moods
	Get angry or wild quickly
	Trouble sleeping
Substances	Tobacco
	Grog

## Results

Surveys were analysed from a total of 842 participants over four communities. The mean (sd) age of the sample was 25.2 (5.3) years and 352 (41.9%) of the sample were male. The mean (sd) of the primary outcome was 8.1 (1.9), kurtosis 2.4, and skewness -0.7. The model had good fit statistics, *χ/df* = 2.69, CFI = 0.95 and RMSEA = 0.045.

Table 3 presents the means, standard deviations and reliability estimates for the items that comprise each latent concept. All construct reliabilities were acceptably high. Cronbach’s alpha is high for ‘importance of culture’ (0.98) indicating that some latent items could be redundant. However given the paucity of validated cultural indicators in this context and our exploration of these in subsequent analyses (in separate reports), we retained this measure in order to develop and refine cultural indicators. While this does not change the results, the high correlation between latent items confirms how closely the communities view these attributes as reflecting culture (Fig. 3).

**Table 3:** Means, standard deviations and Cronbach alphas for each latent concept

Latent concept	Mean	Standard deviation	Cronbach alpha
Importance of culture	2.88	1.51	0.98
Practice culture	2.59	1.03	0.82
Culture in School	2.60	0.93	0.76
Aboriginal literacy	2.45	1.56	0.96
Empowerment	2.39	0.58	0.84
Community	2.96	0.96	0.85
Motivations for education	0.30	0.34	0.92
English literacy and numeracy	2.23	0.73	0.91
Work	1.33	1.00	0.80
General health	3.23	0.67	0.84
Social and emotional wellbeing	2.93	0.94	0.82
Substances	1.97	0.87	0.82

Table 4 displays the bivariate correlations between the latent traits. Most were highly significant. Covariances between variables that were non-significant (corresponding to correlation whose absolute value is below 0.1) were removed in the final statistical model. All cultural variables correlated highly with each other, and with empowerment and community (Table 4). While ‘importance of culture’, ‘practice culture’ and ‘culture in school’ all correlated negatively with ‘English literacy and numeracy’, they all correlated positively with ‘Aboriginal literacy’ which in turn correlated positively with ‘English literacy and numeracy’, suggesting cultural factors have indirect impacts on education through Aboriginal literacy. In depth analysis of these mediated interrelationships are reported separately. Empowerment correlated highly with all education and work variables. There is more variation between health variables suggesting more complex pathways. ‘Substances’ (measured positively with higher scores indicating less use of tobacco and alcohol) was linked with better outcomes across culture, education and work.

**Table 4:** Correlations^a^ between variables

	Importance of culture	Practice culture	Culture in school	Aboriginal literacy	Empowerment	Community	Motivations for education	English literacy and numeracy	Work	General health	Social and emotional wellbeing
Practice culture	0.64***	-									
Culture in school	0.56***	0.68***	-								
Aboriginal literacy	0.18***	0.31***	0.26***	-							
Empowerment	0.11**	0.21***	0.21***	0.17***	-						
Community	0.32***	0.37***	0.46***	0.21***	0.39***	-					
Motivations for Education	0.13***	0.12**	0.17***	0.09*	0.21***	0.07	-				
English literacy and numeracy	-0.21***	-0.14***	-0.11*	0.36***	0.30***	-0.02	0.15***	-			
Work	0.22***	0.15***	0.22***	0.16***	0.32***	0.04	0.26***	0.38***	-		
General health	-0.07	0.01	-0.11**	-0.05	0.07	-0.04	-0.10*	0.02	-0.14***	-	
Social and emotional wellbeing	-0.18***	-0.10*	-0.15***	0.00	-0.02	0.01	-0.19***	-0.03	-0.31***	0.21***	-
Substances	0.11**	0.04	0.22***	0.19***	0.06	0.05	0.16***	0.15***	0.23***	-0.05	-0.01

*p* < 0.05, ** *p* < 0.01, *** *p* < 0.001

^a^ Covariances between variables that were with non-significant were removed from the final model

All items have been re-coded in a positive direction to focus on strengths

The statistical model and the estimates of the standardized structural path coefficients are presented in Table 5. Several constructs load directly onto wellbeing. Constructs with the most direct positive impact on wellbeing include – in order of impact – ‘social and emotional wellbeing’, ‘English literacy and numeracy’, ‘Aboriginal literacy’, ‘substances’ (lack thereof), ‘work’ and ‘community’. Other constructs may influence wellbeing through mediated pathways and these will be explored in separate analyses.

**Table 5:** Standardized path coefficients for the statistical model

Latent concept	Standardized regression weights	*p*-value
Importance of culture	-0.06	0.24
Practice culture	0.08	0.24
Culture in school	**-0.16**	**0.03**
Aboriginal literacy	**0.14**	**<0.001**
Empowerment	0.07	0.13
Community	**0.08**	**0.05**
Motivations for education	**-0.11**	**0.004**
English literacy and numeracy	**0.15**	**<0.001**
Work	**0.12**	**0.02**
General health	-0.07	0.06
Social and emotional wellbeing	**0.23**	**<0.001**
Substances	**0.13**	**0.003**

Latent concepts in bold are statistically significant (*P* < 0.05)

Although configural invariance was established as per the fit statistics above, metric (weak) invariance was not, CMIN = 83.4, df = 38, *p* <0.001. Factor loading in each construct were constrained in separate models to examine the degree of heterogeneity between remote and very remote communities and the following constructs were found to be statistically different: empowerment (*p* = 0.006), community (*p* = 0.016), culture in school (*p* = 0.003), Aboriginal literacy (*p* = 0.001), and general health (*p* = 0.02). Although the results are presented with pooled data, further investigation with broader datasets is required to investigate the diversity between groups. Further analysis with a larger dataset is required to explore these differences.

## Discussion

These analyses confirm statistically the holistic nature of wellbeing for Aboriginal people in remote Australia, and the importance of culture, empowerment and community to government priority areas of education, work, health and wellbeing. The evidence reported here demonstrates that all of these factors interplay through both direct and indirect relationships. They all interrelate - they all influence one another and exist as one entity. This suggests that governments can ‘close the gap’ on their priority areas of education, employment, health and wellbeing through policy and programs that build from the Aboriginal priority areas of culture, empowerment and community, such as the Empowered Communities program [41]. As such, the methodology and framework presented here provide a culturally and scientifically valid approach for different groups to work together to develop a shared system of knowledge, values and goals.

International studies confirm the influence of social determinants on health outcomes, particularly noticeable in socially disadvantaged groups such as Aboriginal and Torres Strait Islanders [42, 43]. Our findings confirm that social factors directly influence wellbeing in this group, particularly social and emotional wellbeing, education, work, community and substance use.

Our findings emphasize a key role for culture and empowerment in the sphere of wellbeing. For example, Aboriginal literacy has a direct positive impact on wellbeing and other cultural factors appear to have equally important but less direct impacts on wellbeing. These include learning about culture at school, strong links between the community and school, learning in one’s first language at school (bilingual education), practicing culture through ‘caring for country’ and hunting for food sources, together with the importance of law and ceremony in one’s life. Validation of these indicators confirms the foundational role they play in the wellbeing of Aboriginal people in remote Australia.

Of relevance to remote communities - where people are more likely to live on or near their ancestral lands - is the prominence amongst these validated cultural indicators of measures relating to connection to language, land, law and ceremony. The interplay and role of these factors may play out differently in more urban communities where these connections can be strained. Our analysis suggests some indicators behave differently in relation to remoteness amongst our cohort. Further investigation is therefore required to better understand the impacts of remoteness, and cultural and geographic diversity to the holistic system of wellbeing.

The use of cultural indicators is growing in research such as in linking land management roles (‘caring for country’) and health [38, 39] - and in government monitoring [8, 14, 30, 33]. Data extracted from national surveys have also been used to show the importance of cultural attachment for wellbeing based on cultural activities, and use of land and language [44, 45]. Dockery also showed differences in a derived indicator of cultural attachment between Indigenous people living in remote compared with urban Australia [44]. However national datasets are limited in addressing cultural and geographic diversity and further work is therefore required to develop more sophisticated knowledge and indicators to better understand diversity and its impacts [2]. For example, communities in tropical and desert regions can both be considered remote but differ vastly in climate and population density, but also in terms of cultural practices [2, 25, 46].

Similar to the current research, localised approaches have used qualitative research to inform the development of quantitative measures to understand Aboriginal wellbeing [47, 48]. As this body of research grows, it becomes important to corroborate findings across studies, such that localized research providing a ‘depth’ of understanding informs cultural indicators in national studies that address the ‘breadth’ of understanding, to better inform policy and progress.

Importantly the domains of the Interplay Wellbeing Framework that represent priorities identified by Aboriginal people in remote communities - including culture, empowerment and community - all correlated strongly with each other. Their relevance to more government prioritized areas of education, work and health was not only confirmed by the entire model goodness-of-fit statistics, but also through subsequent analysis showing strong relationships between Aboriginal literacy and both English literacy and numeracy, and wellbeing. This suggests that Aboriginal literacy developed through learning about culture in school, and learning in one’s first language in school, is a key stepping stone to achieve success in English literacy and numeracy, and improve wellbeing overall. These interpretations are consistent with the correlation analyses presented here and we will explore them in more depth through mediation analyses as part of structural equation modeling to be reported subsequently.

Strong correlations shown between empowerment with all education and work variables signify a key role for building empowerment to improve outcomes across government priority areas of education and employment, and wellbeing overall. Our findings support an existing model demonstrating the importance of building empowerment based on cultural and spiritual beliefs, to improve wellbeing for Indigenous Australians [49, 50]. Our measures of empowerment are based on self-reported resilience, self-efficacy and identity. The strong correlations reported here between culture, empowerment and community indicators suggest building empowerment is closely linked to strengthening community and culture. Based on our data presented here, strengthening community means building safety, connectedness, trust and respect. Building cultural strengths means fostering connections with language, land, law and ceremony. Analysis also suggests that reduced use of alcohol and tobacco leads to stronger representation in culture, education and work. These interrelationships will be explored in more detail through subsequent mediation analyses.

Aboriginal communities are heterogeneous and our approach addresses this dependence on context through a mixed methods design. However, limitations of this paper include that participants were surveyed from four remote communities only and further data is required to understand how generalizable these findings are nationally. Framework development was guided by our research questions and may have evolved differently for a different purpose. Further limitations include the reliance on self-report and that many items were not normal in distribution. However, we tested the robustness of our results by rerunning the analyses in Stata with robust standard errors and found the same results in terms of statistical significance. Further, objective measures are also limited in addressing the non-tangible and subjective aspects of wellbeing that are recorded here.

Although there are some positive developments in this area [41], Aboriginal wellbeing policy strategies have historically been criticized as ‘top down’, ‘one-size-fits-all’, focusing on disadvantage rather than strengths, and looking at education, employment and health separately rather than at the whole of system level [2]. A lack of genuinely involving Aboriginal and Torres Strait Islander peoples in defining and measuring concepts that shape their lives through government policy has received increasing attention [2, 13]. Further, the cultural diversity represented by Aboriginal and Torres Strait Islander peoples across Australia, and the vastly different life experiences of those living in remote compared with urban places has challenged national policy approaches.

Here we present an integrated model that is innovative in terms of its three primary characteristics: (1) the ‘*shared space*’ approach to working collaboratively across languages and cultural worldviews with the key stakeholder groups; (2) considering these challenges at a whole-of-system level, to understand how different components *Interplay* or work together as part of an interconnected system and (3) ‘*bringing together stories and numbers*’ to represent Aboriginal values in western monitoring systems to inform policy. An integrated research approach such as this is extremely rare in the literature and innovative in its approach and application in the proposed context. The Interplay Wellbeing Framework has since been considered for similar applications in substance use and identity research, and for evaluation of the wellbeing impacts of policies and programs.

The framework and data presented here form a baseline against which longitudinal data are being collected and will be reported in the future as part of a prospective cohort design. The Interplay Wellbeing Framework provides a statistical tool to measure and strengthen wellbeing. It has a web-based representation using tailored data visualisation software, showcasing integrated ‘stories and numbers’ to optimise accessibility to general audiences [51]. The visualization represents statistics such as those presented here together with video stories recorded from Aboriginal community members voicing their perspectives on how areas represented in the framework play out in their lives. Data derived from this integrated approach can illuminate the relative balance of investment necessary to have the most significant wellbeing benefits for Aboriginal people.

### Implications for policy and community

These analyses confirm that wellbeing is holistic and solutions must therefore be considered at the ‘whole of system’ level, meaning they must address all of the different areas that interplay to impact ones wellbeing. It further suggests that Australian Governments can best meet their ‘Closing the Gap’ objectives through policies and programs that are developed with the active participation of Aboriginal people and strengthen culture, empowerment and community.

As part of the shared space approach to knowledge translation, this research has been represented in a variety of output including the interactive Interplay Wellbeing Framework that includes statistics (numbers) and 30+ short video documentaries (stories) [51], a talk at TEDx StKilda [52], conferences, academic publications (in development) and a series of posters to report preliminary outcomes [53].

These outputs were officially launched in November 2016 through a number of events: with policy makers at the Department of Prime Minister and Cabinet; and in each participating community. In addition to informing policy, participating communities have benefited from the shared space approach through their active participation and capacity development through the research, and report confidence of using both the networks and resources built in their future work. Detailed qualitative reports on this process will follow.

Based on subsequent collection of qualitative and quantitative data using a community-level survey, development work is underway to validate the Interplay Wellbeing Framework to evaluate the wellbeing impacts of programs, policies and service delivery.

## Conclusion

These analyses confirm that culture, empowerment and community play key roles in the interplay with education, employment and health, as part of a holistic and quantifiable system of wellbeing. Further it provides an inclusive and empowering method for governments to work together with diverse communities towards the betterment of wellbeing.

Finally, this research has broader implications in the collaborative development and validation – both culturally and scientifically - of the Interplay Wellbeing Framework and accompanying survey, new indicators for culture, empowerment and community, and the knowledge translation process including the interactive data visualization.
